# Effects of Luteolin and Apigenin on Adipogenesis Markers PPARγ and FABP4 and Thermogenesis Marker UCP1 in 3T3-L1 Preadipocyte Cell Line

**DOI:** 10.3390/ijms27010139

**Published:** 2025-12-22

**Authors:** Gülcan Uysal Yeler, Ayşegül Sivaslıoğlu, Tuğba Gülsün, Zeynep Göktaş

**Affiliations:** 1 Department of Nutrition and Dietetics, Faculty of Health Sciences, Hacettepe University, Adnan Saygun Street, No. 32, D Block, 06100 Ankara, Türkiye; gulcan.uysal12@hacettepe.edu.tr (G.U.Y.); aysegul.deliloglu@hacettepe.edu.tr (A.S.); 2 Department of Pharmaceutical Technology, Faculty of Pharmacy, Hacettepe University, Adnan Saygun Street, No. 25-3, 06100 Ankara, Türkiye; tgulsun@hacettepe.edu.tr

**Keywords:** adipocyte, adipogenesis, apigenin, cell differentiation, luteolin

## Abstract

Peroxisome proliferator-activated receptor γ (PPARγ) plays a crucial role in the differentiation and maturation of preadipocytes. PPARγ promotes adipogenesis by inducing the expression of fatty acid-binding protein 4 (FABP4). Uncoupling protein 1 (UCP1) is involved in non-shivering thermogenesis and adipocyte browning. The present study aimed to examine the effects of luteolin and apigenin on the gene expression levels and protein concentrations of PPARγ and FABP4, which are involved in adipogenesis, and their effect on UCP1, a thermogenic protein, in the 3T3-L1 preadipocyte cell line. Luteolin and apigenin were prepared at concentrations of 10, 20, and 40 µM and applied to 3T3-L1 preadipocytes during differentiation and maturation. Gene expression levels were measured by real-time PCR, and protein concentrations were measured by ELISA. It was found that the doses used did not cause cytotoxicity in the cells. Luteolin treatment during differentiation and maturation resulted in a decrease in PPARγ and FABP4 gene expression, although the protein concentrations remained unchanged. Additionally, while luteolin treatment did not significantly alter UCP1 gene expression or protein levels during differentiation, it led to a decrease in UCP1 protein concentration during maturation. Apigenin treatment also tended to decrease PPARγ and FABP4 gene expression compared to the control, although no statistical difference was observed. These results suggest that luteolin and apigenin may have regulatory effects on adipogenesis by modulating PPARγ, FABP4, and UCP1 gene expression.

## 1. Introduction

Obesity is defined as excessive fat accumulation and is a complex chronic disease that adversely affects health. Currently, one in eight people worldwide lives with obesity, and the number of obese individuals continues to rise daily [1,2]. Obesity is characterized by hyperphagia (excessive food intake) and adipose tissue hyperplasia (an increase in adipocyte number) [3].

Adipose tissue is a complex structure composed of endothelial cells, fibroblasts, macrophages, and adipocytes, among other cell types. The predominant cells found in adipose tissue are mature adipocytes [4]. Adipogenesis is the process through which fibroblast-like preadipocytes undergo differentiation and maturation to become fully functional, mature adipocytes [3,4]. There are notable differences in the origin and function of white and brown adipocytes [5]. White adipocytes are primarily responsible for lipid storage, whereas brown adipocytes are responsible for heat production [6]. Brown adipocytes contain multilocular lipid droplets and are rich in uncoupling protein 1 (UCP1)-containing mitochondria [7]. UCP1 is a key regulator of non-shivering thermogenesis [8]. It facilitates heat production by interfering with proton leakage across the mitochondrial membrane during oxidative phosphorylation of fatty acids. This process generates a proton gradient, which usually drives ATP production but in the presence of UCP1, energy is dissipated as heat [9]. Given that brown adipose tissue’s energy-dissipating function may play a role in counteracting obesity, researchers are investigating the browning (or beiging) of white adipocytes as a potential strategy for obesity prevention. Current studies are exploring cold exposure and various nutritional compounds as potential inducers of white adipose tissue browning [10,11,12,13,14,15,16,17].

Both brown and white adipocytes require transcription factors such as peroxisome proliferator-activated receptor γ (PPARγ) and other regulatory proteins, including CCAAT/enhancer-binding proteins (C/EBPs) and signal transducers and activators of transcription (STATs), to transition from pre-adipocytes to mature adipocytes [18]. PPARγ, a nuclear receptor, plays a pivotal role in adipocyte differentiation and regulates the metabolic functions of mature adipocytes. Additionally, PPARγ promotes the expression of adipocyte-specific genes, including adiponectin, leptin, and fatty acid-binding protein 4 (FABP4) [19]. FABP4, traditionally considered a cytosolic fatty acid chaperone, is predominantly expressed in adipocytes and is a marker associated with obesity [20]. Its expression is significantly upregulated during adipogenesis [21].

Flavonoids are plant-derived phenolic compounds that are abundant in fruits and vegetables. Flavones, a subgroup of flavonoids, include flavanols, isoflavonoids, flavonones, and anthocyanins. Luteolin (3,4,5,7-tetra-hydroxy-flavone) and apigenin (4,5,7,trihydroxy-flavone) belong to the flavone subgroup [22].

This study aimed to examine the effect of luteolin and apigenin on the gene expression and protein concentrations of PPARγ and FABP4, two key regulators of adipogenesis, as well as their impact on the UCP1 thermogenesis protein in 3T3-L1 preadipocytes.

## 2. Results

### 2.1. Cell Survival Rate

**Figure 1:**
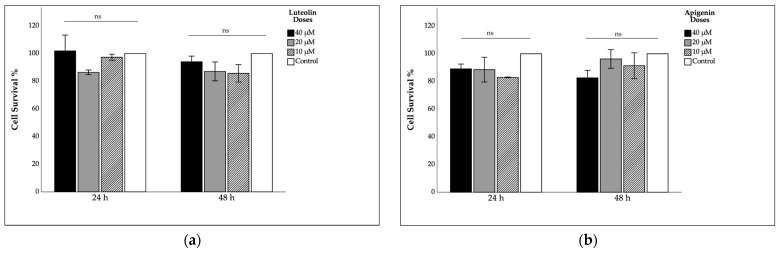
Effect of luteolin and apigenin on cell survival rate: (**a**) luteolin; (**b**) apigenin. Intergroup comparisons were performed using the Bonferroni post hoc test and one-way ANOVA. µM: micromolar; ns: non-significant.

The effects of luteolin and apigenin at concentrations of 10, 20, and 40 µM on cell survival are shown in Figure 1. It was observed that cell viability tended to decrease with 10 and 20 µM luteolin treatment compared to 40 µM treatment and the control, but these decreases were not statistically significant. With 24 h of apigenin treatment, it was shown that the maximum reduction was with the 10 µM treatment, and at 48 h of treatment, the maximum reduction was observed with the 40 µM dose; however, these reductions were not statistically significant. Overall, luteolin and apigenin treatment appear to cause a non-linear decrease that is both dose- and application time-dependent but does not cause cytotoxicity.

### 2.2. Gene Expression Levels

**Figure 2:**
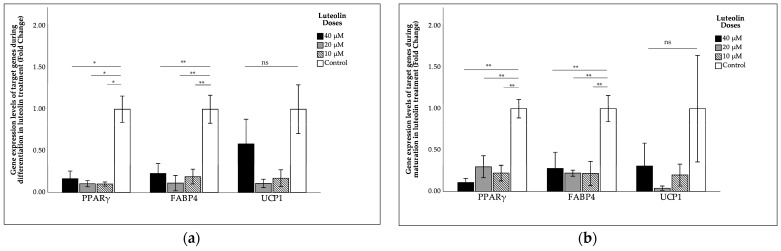
Effects of luteolin on gene expression levels of *PPARγ*, *FABP4*, and *UCP1* during (**a**) differentiation; (**b**) maturation. Intergroup comparisons were performed using the Bonferroni post hoc test and one-way ANOVA. *: *p* < 0.001; **: *p* < 0.05; ns: non-significant. FABP4, fatty acid-binding protein 4; UCP1, uncoupling protein 1; PPARγ, peroxisome proliferator-activated receptor gamma.

The effects of luteolin on *PPARγ*, *FABP4*, and *UCP1* gene expression during differentiation and maturation are shown in Figure 2. The results demonstrated that the decrease in *UCP1* gene expression during differentiation and maturation following luteolin treatment were minimal and statistically non-significant compared to the control group. However, luteolin treatment during differentiation and maturation was found to reduce *PPARγ* and *FABP4* gene expression levels compared to the control.

**Figure 3:**
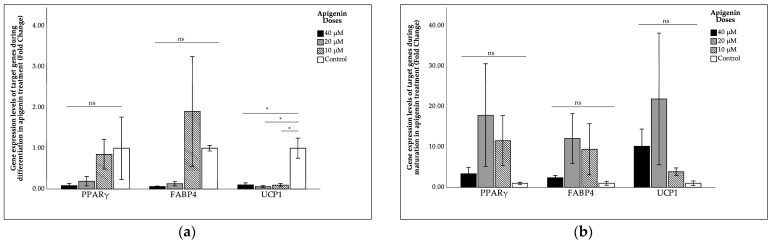
Effects of apigenin on gene expression levels of *PPARγ*, *FABP4*, and *UCP1* during (**a**) differentiation; (**b**) maturation. Intergroup comparisons were performed using the Bonferroni post hoc test and one-way ANOVA. *: *p* < 0.05; ns: non-significant. FABP4, fatty acid-binding protein 4; UCP1, uncoupling protein 1; PPARγ, peroxisome proliferator-activated receptor gamma.

The effects of apigenin on *PPARγ*, *FABP4*, and *UCP1* gene expression during differentiation and maturation are shown in Figure 3. The results showed that the elevations in *PPARγ* and *FABP4* gene expression levels following apigenin treatment during differentiation and maturation were minimal and statistically non-significant compared to the control group. However, apigenin treatment during differentiation led to a significant decrease in *UCP1* gene expression (*p* = 0.02). In contrast, apigenin treatment during maturation tended to increase *UCP1* gene expression, but this increase was not statistically significant.

### 2.3. Protein Concentrations

The protein concentrations of PPARγ, UCP1, and FABP4 after luteolin and apigenin treatment are presented in Table 1. The study revealed that luteolin treatment during differentiation did not cause significant changes in PPARγ, FABP4, and UCP1 protein levels. Similarly, luteolin treatment during maturation did not affect PPARγ and FABP4 protein levels; however, a dose-dependent decline in UCP1 protein concentration was observed.

The findings also indicated that apigenin treatment had no effect on PPARγ, FABP4, and UCP1 protein concentrations during both differentiation and maturation. The only statistically significant change was observed during maturation, where an increase in UCP1 protein levels was detected compared to the levels during differentiation.

### 2.4. Triglyceride Levels and Lipid Accumulation

**Figure 4:**
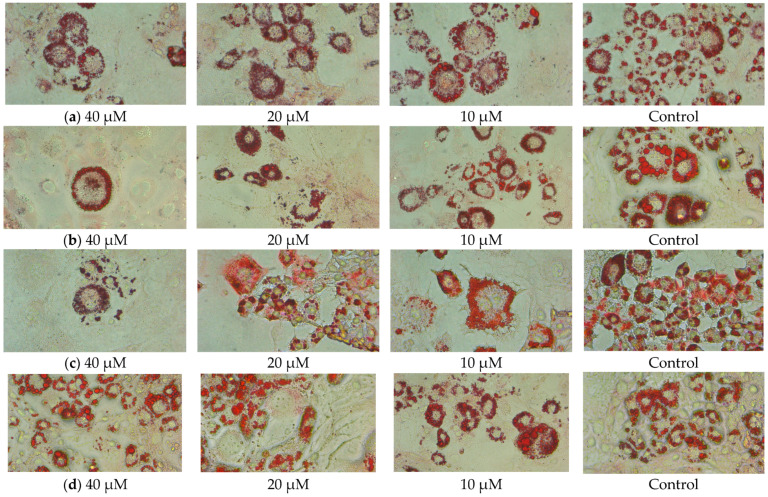
Oil Red O staining. (**a**) Luteolin at differentiation; (**b**) luteolin at maturation; (**c**) apigenin at differentiation; (**d**) apigenin at maturation. µM: micromolar.

No statistically significant difference was observed in intracellular triglyceride (TG) levels between different doses of luteolin and apigenin treatments given during both differentiation and maturation (Table 2). Figure 4 shows images of cells stained with Oil Red O. The cells are surrounded by lipid droplets and appear red with the dye.

## 3. Discussion

Research on obesity is increasingly focusing on determining whether the browning or beiging of white adipocytes can be induced, with various nutrients or phytochemicals being studied for this purpose. The present study investigated the effects of luteolin and apigenin on both the gene expression and protein levels of the adipogenic proteins PPARγ and FABP4 and thermogenic protein UCP1 in 3T3-L1 preadipocytes.

PPARγ plays a central role in activating adipogenesis and is upregulated early in the differentiation of preadipocytes into mature adipocytes [23]. Luteolin has been reported to suppress preadipocyte differentiation, particularly in the early stages of adipogenesis [24]. In a study where luteolin was administered to 3T3-L1 cells, luteolin caused a decrease in *PPARγ* gene expression and inhibition of PPARγ activity at the hormone-mixture-induced differentiation stage [24]. Similarly, luteolin administered during differentiation downregulated *PPARγ* and *FABP4* gene expression in a dose-independent manner [25]. Other studies have also confirmed similar decreases in PPARγ and FABP4 expression following luteolin treatment during differentiation [26,27]. Zhao et al. reported that both luteolin and apigenin downregulated adipogenesis-related genes, including *PPARγ* and *FABP4*, with luteolin being more effective [28]. Similarly, in another study, apigenin treatment caused a decrease in *PPARγ* expression [29]. However, another study using apigenin reported a reduction in *PPARγ* gene expression but no change in *FABP4* expression [30]. Hong et al. showed that apigenin reduced lipid accumulation by downregulating *PPARγ* expression [31]. Unlike previous studies, it was found that apigenin treatment during differentiation and maturation of human mesenchymal cell-derived adipose cells did not cause changes in *PPARγ* expression [32]. In the present study, luteolin treatment during differentiation and maturation resulted in a reduction in PPARγ and FABP4 gene expression, while the protein concentrations remained unchanged. Apigenin treatment, on the other hand, decreased *PPARγ* and *FABP4* gene expression, but this reduction was not statistically significant. During adipogenesis, changes occur in transcription and translation regulation and these changes vary over time [33]. The fact that the changes seen in gene expression were not seen in the protein concentrations may be related to this. In addition, the fact that PPARγ has different functions during adipogenesis may also explain the lack of change in its protein levels [34].

UCP1 is a key protein involved in thermogenesis and is primarily found in brown adipose tissue and beige adipocytes. The induction of beige adipocytes typically requires external stimuli, such as cold exposure, exercise, or bioactive compounds [35]. Liu et al. observed that luteolin treatment of fully differentiated 3T3-L1 cells resulted in increased *UCP1* expression [25]. In high-fat diet-fed mice, luteolin enhanced the thermogenic program by increasing *UCP1* expression in both brown and subcutaneous adipose tissue [36]. However, in this study, luteolin treatment during maturation led to a decrease in the UCP1 protein concentration, while no significant effects were observed during differentiation. Regarding apigenin, Sun et al. reported that apigenin administration in mice led to the downregulation of PPARγ, FABP4, and LPL in subcutaneous and epididymis adipose tissues, but no significant effects were observed in brown adipose tissue. However, *UCP1* expression increased in brown and subcutaneous adipose tissue following apigenin treatment [37]. Similarly, a combination of apigenin and resveratrol resulted in increased *UCP1* expression [38]. In the present study, apigenin treatment during both differentiation and maturation did not significantly alter *UCP1* expression levels.

In a study, luteolin treatment of 3T3-L1 cells during differentiation was found to decrease the intracellular lipid content in a dose-dependent manner after 10 days [25]. In another study, luteolin and apigenin decreased intracellular TG levels 9 days after differentiation [27]. Apigenin treatment of human mesenchymal stem cells during differentiation to adipose cells had no effect on intracellular TG levels; it was not effective at low doses in mature cells but caused a decrease at high doses [32]. Similar to our study, another study found a dose-dependent decrease in lipid droplets according to Oil Red O staining results with both luteolin and apigenin treatment [27]. According to the results of this study, the lack of effect of apigenin treatment on the expression of adipogenic genes supports the unchanged TG content. Also, it has been reported that the effect on lipid reduction cannot be definitely attributed to anti-adipogenic activity due to its effect on 3T3-L1 cell viability [30].

In conclusion, this study examined the effects of luteolin and apigenin flavones on the adipogenic markers PPARγ and FABP4 and the thermogenic marker UCP1 in 3T3-L1 preadipocytes. Luteolin treatment resulted in a significant reduction in *PPARγ* and *FABP4* gene expression during both differentiation and maturation. In contrast, apigenin treatment did not induce significant changes in the expression of these genes during either phase.

Unlike most previous studies, this research investigated phytochemical effects during both differentiation and maturation after normal differentiation was completed. Adipogenesis and thermogenesis involve many pathways. According to the results of this study, luteolin and apigenin have detectable effects, even if they are weak. Future in vitro and in vivo studies are necessary to further clarify the molecular mechanisms underlying the adipogenic and thermogenic effects of luteolin and apigenin.

## 4. Materials and Methods

### 4.1. Cell Culture and Treatment

3T3-L1 preadipocyte cells were obtained from the American Type Tissue Collection (ATCC). Frozen cells were thawed and then seeded in T25 flasks. The culture medium was Dulbecco’s Modified Eagle Medium (DMEM; Diagnovum, Greifswald, Germany) with 10% Fetal Bovine Serum (FBS; Diagnovum, D154, Germany) and 1% penicillin/streptomycin (Diagnovum, D910, Germany). Cells were incubated at 37 °C in a 5% CO_2_ incubator. Once the cells reached 90% confluence, they were passaged into 12-well plates. Differentiation of 3T3-L1 preadipocytes was induced by supplementing the medium with 3-isobutyl-1-methylxanthine (IBMX, 0.5 M; Sigma, Burlington, MA, USA), dexamethasone (DEX, 1 µM; Item No. 11015, Cayman, Ann Arbor, MI, USA), and insulin (10 µg/mL, insulin from bovine pancreas; I6634, Sigma, USA). To evaluate the effects of luteolin and apigenin on preadipocyte differentiation, 3T3-L1 cells were treated with luteolin or apigenin (10, 20, and 40 µM) during the differentiation process, with fresh treatment applied with every medium change. Additionally, another set of cells was treated with luteolin or apigenin for 48 h post-maturation. The control group received sterile phosphate-buffered saline (PBS; 806552, Sigma, USA).

Luteolin (>98% TCI CHEM T2682 Cas No. 491-70-3) and apigenin (>98% TCI CHEM A1514 Cas No. 520-36-5) were purchased TCI Chem Europe (Tokyo, Japan). Luteolin and apigenin were prepared as 1000 µM stock solutions by dissolving the compounds in dimethyl sulfoxide (DMSO; Cas No. 67-68-5, Sangon Biotech, Shanghai, China), which were diluted with sterile PBS to final concentrations of 10, 20, and 40 µM. These concentrations were selected based on the literature [27,30,39]. Treatments were applied to each well after filtration through a sterile filter, with sterile PBS serving as the control. Three biological replicates were collected and each of these biological replicates was run in duplicate in the experiments.

### 4.2. Cell Survival Assay

Undifferentiated 3T3-L1 cells were used in the Cell Survival Assay. Cell viability analysis was performed using the Elabscience^®^ MTT Assay Kit (E-CK-A341, Houston, TX, USA) according to the manufacturer’s protocol. In summary, cell suspensions were seeded into a 96-well plate and incubated for 24 h. Luteolin (10 µL), apigenin (10 µL), or PBS (10 µL) was added to the wells, and the plates were incubated at 37 °C in a 5% CO_2_ incubator for 24 or 48 h. At the end of the incubation period, the MTT working solution was added to each well and incubated for 4 h. The supernatant was then discarded, dimethyl sulfoxide (DMSO) was added, and absorbance was measured at 570 nm.

### 4.3. Measuring Gene Expression Levels

Total RNA was extracted using the One-Step RNA Reagent (BioBasic, Markham, ON, Canada, Cat. No. BS410A) according to the manufacturer’s protocol. The RNA extraction protocol included phase separation, RNA precipitation, washing, and re-dissolving steps. At the end of the procedure, the RNA concentration of the samples was measured using a BioSpec nano-spectrophotometer (Shimadzu, Kyoto, Japan). RNA was then reverse transcribed into complementary DNA (cDNA) using the OneScript^®^ Plus cDNA Synthesis Kit (ABM Good, Cat. No. G236, Delray Beach, FL, USA). Briefly, reverse transcription buffer, dNTP, primers, the total RNA sample, OneScript Plus RTase, and nuclease-free water were mixed on ice and incubated in a Thermal Cycler (Thermo Scientific, Waltham, MA, USA, PIKO96) at 50–55 °C for 15 min. The reaction was terminated by heating at 85 °C for 5 min. Quantification of gene expression was performed using the BlastaqTM 2X PCR Master Mix kit (ABM Good, Cat. No. G891), and qPCR analysis was conducted using a LightCycler 480 (Roche Company, Basel, Switzerland). The qPCR protocol consisted of denaturation at 95 °C for 3 min, followed by 45 cycles of denaturation at 95 °C for 15 s and annealing/extension at 60 °C for 1 min. Gene expression levels were calculated using the ΔΔCt method. The expression levels of *PPARγ*, *FABP4*, and *UCP1* were normalized to those of *B-actin*. The primer sequences used for the gene expression analysis are listed in Table 3.

### 4.4. Protein Extraction and Measurement of Protein Levels

Total protein was extracted in three steps: precipitation, washing, and solubilization. The concentrations of PPARγ, FABP4, and UCP1 proteins were analyzed using the sandwich enzyme immunoassay method with ELISA kits (USCN, Wuhan, China) following the manufacturer’s instructions. The enzyme–substrate reaction induced a color change, which was measured at 450 nm using a spectrophotometer. Protein concentrations were determined by comparing the optical density (OD) of the samples to a standard curve.

### 4.5. Measurement of Triglyceride Levels

The Triglyceride Colorimetric Assay Kit (Elabscience Biotechnology Inc., Houston, TX, USA) was used to measure the triglyceride concentration in each sample. The concentration was calculated using the equation provided in the kit’s protocol. Triglyceride levels in 3T3−L1 adipocytes were expressed as a percentage of the control values.

### 4.6. Oil Red O Staining

Cells were stained with Oil Red O to assess lipid accumulation. After aspirating the culture medium and washing the plate with PBS, cells were fixed with 10% formalin for 1 h. Following fixation, the formalin was removed, and the cells were washed with distilled water before adding 60% isopropanol. The prepared Oil Red O (Cas No. 1320-06-5, Acros Organics, Geel, Belgium) working solution was then added, and the cells were incubated for 10–20 min. After washing with distilled water, the excess dye was removed, and the cells were imaged under a microscope (Flexacam C3, Leica Microsystems, Heerbrugg, Switzerland).

### 4.7. Statistical Analysis

Statistical analysis was performed using SPSS 22.0 (IBM Corp., Armonk, NY, USA). One-way ANOVA was performed, followed by multiple comparison tests using the Mann–Whitney U test to compare the treatment results with those of the controls. Additionally, LSD or Games–Howell tests were used to compare difference between the groups. Data are presented as numbers and percentages or means ± standard errors (SEs). A *p*-value < 0.05 was considered statistically significant.

## Figures and Tables

**Table 1:** Concentrations of analyzed proteins ^1,2,3^.

		Differentiation			Maturation		
Phytochemical	Doses	PPARγ	FABP4	UCP1	PPARγ	FABP4	UCP1
Luteolin	40 µM	0.73 ± 0.022	0.37 ± 0.001	0.37 ± 0.004	0.47 ± 0.020	0.20 ± 0.056	0.87 ± 0.038 ^a^
	20 µM	0.72 ± 0.008	0.37 ± 0.001	0.37 ± 0.0002	0.48 ± 0.086	0.20 ± 0.001	0.87 ± 0.023 ^b^
	10 µM	0.70 ± 0.005	0.36 ± 0.001	0.37 ± 0.0004	0.68 ± 0.172	0.20 ± 0.014	1.27 ± 0.075 ^a,b^
	Control	0.74 ± 0.007	0.37 ± 0.001	0.37 ± 0.001	0.48 ± 0.016	0.22 ± 0.022	1.19 ± 0.068 ^a,b^
		*p* > 0.05	*p* > 0.05	*p* > 0.05	*p* > 0.05	*p* > 0.05	***p* = 0.001**
Apigenin	40 µM	0.74 ± 0.025	0.37 ± 0.003	0.37 ± 0.002	0.80 ± 0.095	0.39 ± 0.025	1.56 ± 0.273
	20 µM	0.71 ± 0.011	0.37 ± 0.001	0.37 ± 0.001	0.77 ± 0.018	0.37 ± 0.022	1.39 ± 0.145
	10 µM	0.76 ± 0.030	0.36 ± 0.001	0.37 ± 0.003	0.69 ± 0.111	0.32 ± 0.059	1.74 ± 0.332
	Control	0.72 ± 0.006	0.36 ± 0.001	0.37 ± 0.003	0.66 ± 0.078	0.31 ± 0.021	1.48 ± 0.299
		*p* > 0.05	*p* > 0.05	*p* > 0.05	*p* > 0.05	*p* > 0.05	*p* > 0.05

^1^ Data were analyzed by one-way ANOVA. ^2^ Data are presented as mean ± SE, ^3^ Means with a common letter are statistically significantly different (*p* < 0.05). The *p*-value in bold is statistically significant.

**Table 2:** Triglyceride contents of cells ^1,2^.

	Dose	Differentiation	Maturation
Luteolin	40 µM	5.31 ± 0.703	73.56 ± 10.641
	20 µM	7.35 ± 0.468	85.43 ± 0.986
	10 µM	33.64 ± 15.201	52.85 ± 23.666
	Control	5.13 ± 1.391	66.51 ± 7.068
		*p* > 0.05	*p* > 0.05
Apigenin	40 µM	21.69 ± 12.409	8.94 ± 2.837
	20 µM	2.48 ± 1.151	19.03 ± 11.601
	10 µM	6.81 ± 1.566	40.986 ± 30.631
	Control	17.09 ± 13.578	11.60 ± 0.898
		*p* > 0.05	*p* > 0.05

^1^ Data were analyzed by one-way ANOVA. ^2^ Data are presented as mean ± SE.

**Table 3:** Sequences of primers.

Gene	Forward Primer	Reverse Primer
*B-actin*	CCTGTGCTGCTCACCGAGGC	GACCCCGTCTCTCCGGAGTCCATC
*PPARγ*	GTACTGTCGGTTTCAGAAGTGCC	ATCTCCGCCAACAGCTTCTCCT
*FABP4*	TGAAATCACCGCAGACGACAGG	GCTTGTCACCATCTCGTTTTCTC
*UCP1*	CCTGCCTCTCTCGGAAACAA	GTAGCGGGGTTTGATCCCAT

*FABP4*, fatty acid-binding protein 4; *UCP1*, uncoupling protein 1; *PPARγ*, peroxisome proliferator-activated receptor gamma.

## Data Availability

The raw data supporting the conclusions of this article will be made available by the authors on request.

## Abbreviations

The following abbreviations are used in this manuscript:

ATCC	American Type Culture Collection
cDNA	complementary DNA
Ct	cycle threshold
C/EBPs	CCAAT/enhancer-binding proteins
DEPC	diethyl pyrocarbonate
DEX	dexamethasone
DMEM	Dulbecco’s Modified Eagle Medium
DMSO	dimethyl sulfoxide
ELISA	enzyme-linked immunosorbent assay
FABP4	fatty acid-binding protein 4
FBS	Fetal Bovine Serum
IBMX	3-isobutyl-1-methylxanthine
MTT	3-(4,5-dimethylthiazolyl-2)-2,5-diphenyltetrazolium bromide assay
nm	nanometer
OD	optical density
PBS	phosphate-buffered saline
PCR	polymerase chain reaction
PPARγ	Peroxisome Proliferator-Activated Receptor γ
STATs	signal transducers and activators of transcription
SE	standard error
UCP1	uncoupling protein 1
TG	triglyceride
µM	micromolar
